# Prediction of Liver Weight Recovery by an Integrated Metabolomics and Machine Learning Approach After 2/3 Partial Hepatectomy

**DOI:** 10.3389/fphar.2021.760474

**Published:** 2021-11-30

**Authors:** Runbin Sun, Haokai Zhao, Shuzhen Huang, Ran Zhang, Zhenyao Lu, Sijia Li, Guangji Wang, Jiye Aa, Yuan Xie

**Affiliations:** ^1^ Jiangsu Province Key Laboratory of Drug Metabolism and Pharmacokinetics, State Key Laboratory of Natural Medicines, China Pharmaceutical University, Nanjing, China; ^2^ Phase I Clinical Trials Unit, Nanjing University Medical School Affiliated Drum Tower Hospital, Nanjing, China

**Keywords:** liver regeneration, partial hepatectomy, metabolomics, machine learning, GC/MS

## Abstract

Liver has an ability to regenerate itself in mammals, whereas the mechanism has not been fully explained. Here we used a GC/MS-based metabolomic method to profile the dynamic endogenous metabolic change in the serum of C57BL/6J mice at different times after 2/3 partial hepatectomy (PHx), and nine machine learning methods including Least Absolute Shrinkage and Selection Operator Regression (LASSO), Partial Least Squares Regression (PLS), Principal Components Regression (PCR), k-Nearest Neighbors (KNN), Support Vector Machines (SVM), Random Forest (RF), eXtreme Gradient Boosting (xgbDART), Neural Network (NNET) and Bayesian Regularized Neural Network (BRNN) were used for regression between the liver index and metabolomic data at different stages of liver regeneration. We found a tree-based random forest method that had the minimum average Mean Absolute Error (MAE), Root Mean Squared Error (RMSE) and the maximum R square (R^2^) and is time-saving. Furthermore, variable of importance in the project (VIP) analysis of RF method was performed and metabolites with VIP ranked top 20 were selected as the most critical metabolites contributing to the model. Ornithine, phenylalanine, 2-hydroxybutyric acid, lysine, etc. were chosen as the most important metabolites which had strong correlations with the liver index. Further pathway analysis found Arginine biosynthesis, Pantothenate and CoA biosynthesis, Galactose metabolism, Valine, leucine and isoleucine degradation were the most influenced pathways. In summary, several amino acid metabolic pathways and glucose metabolism pathway were dynamically changed during liver regeneration. The RF method showed advantages for predicting the liver index after PHx over other machine learning methods used and a metabolic clock containing four metabolites is established to predict the liver index during liver regeneration.

## Introduction

The liver is the largest internal solid organ (by mass) and has various essential functions for body homeostasis, including digestion, balancing glucose and storing glycogen, regulating blood amino acids, carrying away wastes, detoxifying chemicals, and metabolizing drugs. The liver has a mysterious ability to regenerate. It is the only organ that can regenerate itself to 100% of original weight in mammals (Miyaoka and Miyajima, 2013; Michalopoulos and Bhushan, 2020). It is known that the liver can restore to its original weight from as little as 25% of the original liver mass to guarantee the stability of liver weight about body weight. Based on this feature of the liver, partial hepatectomy (PHx) is widely used in the clinic for liver trauma, intrahepatic gallstones, hepatic cyst, hepatic neoplasms (both benign and malignant), and liver transplantation (Orcutt and Anaya, 2018; O'Grady, 2000; Xia et al., 2014; Nuzzo et al., 2008). Liver regeneration is a highly complex process. Different types of cells and many signaling pathways are involved, including hepatocyte proliferation, reprogramming of extracellular matrix, inflammation, immune and metabolic regulation, etc. (Preziosi and Monga, 2017; Michalopoulos and Bhushan, 2020).

It is important to obtain accurate liver weight for major hepatic resection and living donor liver transplantation. Simply, the total liver volume can be predicted based on body surface area and body weight (Vauthey et al., 2002). However, this method cannot be used to measure liver volume after liver resection. Imaging-based liver volumetric methods include anatomical structure imaging method and functional imaging method. Anatomical structure imaging includes computed tomography (CT) (Ogasawara et al., 1995; Alonso-Torres et al., 2005; Lim et al., 2014; Kim et al., 2019), magnetic resonance imaging (MRI) (Hockings et al., 2002; Sahin et al., 2003; Inderbitzin et al., 2004), ultrasonography (Kitajima et al., 2008; Kasuya et al., 2011), and functional imaging including single-photon emission computed tomography (SPECT) (De Graaf et al., 2008; Stinauer et al., 2012; Yoshida et al., 2014). These methods have shown reliable liver volume measurements and have been widely utilized to evaluate postoperative liver regeneration and assess liver function recovery (Bassignani et al., 2001; Zamboni et al., 2008; de Graaf et al., 2010; Spira et al., 2012). These image-based evaluation methods can achieve the liver weight and the shape of the liver, and functional-based image methods can further evaluate the liver function. However, these methods have a certain degree of error and overestimate the actual liver volume (D'Onofrio et al., 2014). There still remains an urgent need to develop a new method to evaluate liver regeneration and liver function after PHx.

Several non-image methods for liver volumetry have been developed. From a systemic biology view, the microarray data of rat liver during regeneration and the adaptive logistic regression identified M6PR→IGF2R and MCM5→STAT1 pathways as biomarkers for liver regeneration (Chen et al., 2016). Metabolomics is the profile of endogenous small molecules. It is widely used in the early detection of hepatocellular carcinoma (Zhang et al., 2013; Safaei et al., 2016), identification of subtypes and different stages of non-alcoholic steatohepatitis (Alonso et al., 2017; Dong et al., 2017), investigation of hepatitis virus infection (Du Preez and Sithebe, 2013; Huang et al., 2016; Naggie et al., 2020), prediction of and identification of drug-induced liver injury (Xie et al., 2019), and reveal the mode of action of natural products in the treatment of liver disease (Beyoğlu and Idle, 2020). The metabolomics technique is used for liver transplantation to discover biomarkers associated with donor-recipient matching and early allograft dysfunction (Cortes et al., 2014; Faitot et al., 2018). Specifically, bile salt and triglyceride levels are proposed to be early predictors of liver volume and functional increase after liver resection (Hoekstra et al., 2012a; Hoekstra et al., 2012b). The hepatic ratio of phosphatidylcholine to phosphatidylethanolamine is also a survival predictor following partial hepatectomy (Ling et al., 2012). Hyaluronic acid is metabolized by liver sinusoid endothelial cells. Its level can be used to evaluate functional liver reserve after liver resection and prediction of complications associated with liver resection (Nanashima et al., 2001; Nanashima et al., 2004). The L-[1–^13^C]Methionine breath test and the production of ^13^CO_2_ are considered valuable indicators for evaluating liver regeneration (Ishii et al., 2001). These biomarker-based methods can predict the regeneration of the liver as well as liver function recovery.

Several models have been proposed to characterize the process of liver regeneration. A liver growth model based on general growth law has been introduced to accurately predict liver transplants’ growth (Shestopaloff and Sbalzarini, 2014). Furchtgott et al. developed a mathematical model of rat liver regeneration based on the interplay of cytokines and growth factors, and Periwal et al. further transferred this model to humans (Furchtgott et al., 2009; Periwal et al., 2014). These studies used a single approach and are usually limited by moderate accuracy. Machine learning is a subset of artificial intelligence used for clinical diagnostics, prognosis prediction, precision treatments, health monitoring, and drug discovery and development (Vamathevan et al., 2019; Goecks et al., 2020). Machine learning approaches have large flexibility and are free from prior assumptions, and they are particularly suitable for datasets with few observations and many variables, especially for omics data. Traditional statistical methods aim to infer relationships between variables, while machine learning algorithms focus on making predictions as accurate as possible even though some of them are difficult to interpret. Machine learning disentangles the complex relationships between numerous variables of omics studies in determining their effect on the main outcome (Rajula et al., 2020). However, there is no study about predicting the liver index after PHx by integrating metabolomics and machine learning algorithms in our knowledge. Here we use nine machine learning methods including Least Absolute Shrinkage and Selection Operator Regression (LASSO), Partial Least Squares Regression (PLS), Principal Components Regression (PCR), k-Nearest Neighbors (KNN), Support Vector Machines (SVM), Random Forest (RF), eXtreme Gradient Boosting (xgbDART), Neural Network (NNET), and Bayesian Regularized Neural Networks (BRNN) to select the best regression model between the liver index and metabolomics data from serum, discover the main metabolic pathways during liver regeneration, and finally establish a prediction model with a metabolite set to predict the liver index during liver regeneration.

## Materials and Methods

### Chemicals

Methanol (chromatography grade), n-Heptane (chromatography grade), methoxyamine, pyridine, and N-methyl-N-trimethylsilyl-trifluoroacetamide+1% trimethylchlorosilane (MSTFA+1%TMCS) were purchased from Merck KGaA (Darmstadt, Germany). Stable-isotope-labeled [^13^C_2_]-myristic acid was purchased from Cambridge Isotope Laboratories (Andover, MA, United States).

### Animal Studies

**FIGURE 1:**
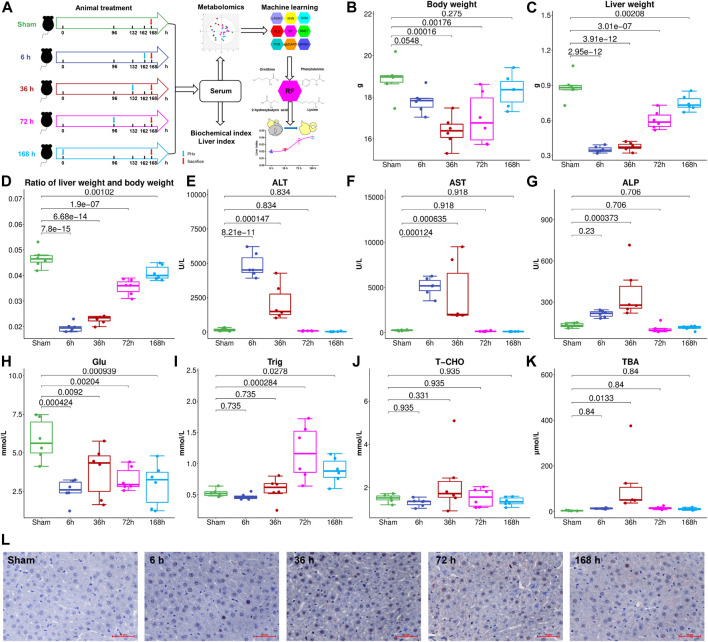
**(A)**, Flowchart of the animal experiment. Liver weight **(B)**, Body weight **(C)**, and liver index **(D)** change at different stages after 2/3 PHx. Liver function index, alanine aminotransferase (ALT) **(E)**, aspartate aminotransferase (AST) **(F)**, and alkaline phosphatase (AKP) **(G)** were measured for the Sham group and 6, 36, 72, and 168 h after PHx, serum glucose **(H)**, triglyceride **(I)**, total cholesterol **(J)**, and total bile acids (TBA) **(K)** were measured. **(L)**, PCNA expression in the livers of mice before and after PHx.

Thirty male C57BL/6J mice (5 weeks old, purchased from Changzhou Cavens Laboratory Animal Co., Changzhou, China) were housed under a 12 h light/12 h dark condition (lights on at 6:00 and lights off at 18:00). All animal care and experimental procedures protocols were approved by the Animal Ethics Committee of China Pharmaceutical University (2018-DMPK-12-06). All mice were fed with a standard chow diet (AIN-93M, Trophic Animal Feed High-Tech Co., Ltd, Nantong, China) and tap water *ad libitum* for 1 week to acclimate the environment. The mice were divided into five groups (n = 6), Sham group (Sham), 6 h after PHx group (6 h), 36 h after PHx group (36 h), 72 h after PHx group (72 h), and 168 h after PHx group (168 h). The mice were anesthetized with isofluorane when doing the PHx surgery. For the Sham group, the abdominal cavity was opened without cutting the liver and then sewed; for PHx groups, the left lateral and median liver lobes, including gall bladder, were resected according to the procedure in literature. The mice were sacrificed 0 h (Sham group), 6, 36, 72 and 168 h after PHx. At the time of sacrifice, mice were weighed and anesthetized by avertin; the whole blood was centrifuged at 8,000 rpm for 5 min to get the serum and was stored at −80°C for further analysis. Livers were harvested and weighed, and the liver index was calculated (liver weight/body weight). The proliferative cell nuclear antigen (PCNA) expression was measured, and images were collected using an inverted microscope (Leica DMI 3000B, Germany). A flowchart of the animal experiment is shown in Figure 1A.

### Measurement of Serum Biochemical Index

Levels of serum glucose, triglyceride, cholesterol, total bile acids (TBA), alanine aminotransferase (ALT), aspartate aminotransferase (AST), and alkaline phosphatase (AKP) were measured using kits purchased from Nanjing Jiancheng Bioengineering Institute (Nanjing, China) according to the manufacturer’s instructions.

### Sample Preparation for GC/MS and Compound Identification

The metabolites in serum were profiled by a GC/MS-based metabolomics method as previously reported (A et al., 2005). Briefly, 50 μL of serum was extracted with 200 μL of methanol containing 5 μg/ml [^13^C_2_]-myristic acid; after oximation and derivatization, 0.5 μL of the sample were injected into a SHIMADZU QP2010Ultra/SE GC/MS system (Kyoto, Japan) with an RTx-5MS fused silica capillary column (30 m × 0.25 mm ID, J&W Scientific, United States). The raw data acquired were processed by GCMSSolution (version 4.11). The metabolites were identified using NIST 14 (National Institute of Standards and Technology, Gaithersburg, MD, United States), Wiley 9 (Wiley–VCH Verlag GmbH & Co KGaA, Weinheim, Germany), and an in-house mass spectra library database (A et al., 2005; Sun et al., 2019).

### The Regression of Liver Index and Metabolites by Nine Machine Learning Methods

PCA was performed for dimension reduction using SIMCA-P 13.0 software (Umetrices, Umeå, Sweden). Nine machine learning methods including LASSO, PLS, PCR, KNN, SVM, RF, xgbDART, NNET, and BRNN were used for regression between the liver index and metabolites. The code used was shown in Supplementary Data Sheet S2. Models were evaluated by the parameters, including the Mean Absolute Error (MAE), the Root Mean Squared Error (RMSE), and R square (R^2^). All of the machine learning methods were performed and tuned using the “caret” package in the R project (version 3.6.3). Variable importance in the projection (VIP) analysis was used to evaluate metabolites’ contribution to the model.

### Pathway Analysis

Metabolomics pathway analysis of the metabolites with VIP >1 was carried out using MetaboAnalyst (www.metaboanalyst.ca). Hypergeometric test for over-representation analysis and relative-betweenness centrality for pathway topology analysis was selected, and Mus musculus (KEGG) library was chosen.

### Selection of Metabolite Set for the Prediction of the Liver Index

Correlation coefficients between liver index, ALT, and metabolites at different time points were calculated. To further evaluate the RF method’s ability to predict the liver index after 2/3 PHx, the dataset was split into the training set and testing set (5:1). The metabolite with the most significant VIP value, the metabolites ranked top 4, 8, 12, 20, 40, 59 and the whole dataset without one metabolite whose VIP is 0 (Supplementary Data Sheet S1) was further used to train the RF model and predict the liver index in the testing set, and their performance was also compared. Models were evaluated by the parameters including MAE, RMSE, and R^2^.

### Statistical Analysis

For statistical analysis of MAE, RMSE, and R^2^ in each model, Kruskal–Wallis Test followed by Wilcox test was used; for statistical analysis of metabolites among groups, One-way ANOVA followed by Fisher’s LSD multiple comparison test and corrected by the Benjamini-Hochberg method to control the False Discovery Rate (FDR) was conducted by R project (version 3.6.3). The correlation coefficients were calculated by the “corrplot” package in the R project. *p* < 0.05 was considered statistically different.

## Results

### Regeneration of Liver After 2/3 Partial Hepatectomy

To investigate liver regeneration progress and the associated metabolic change after partial hepatectomy, 2/3 PHx in C57BL/6J mice was performed and samples were collected at five time points (Sham group, 6, 36, 72, and 168 h after PHx, the total sample size is 30). The liver index was calculated using liver weight and body weight. The remaining liver exhibited an elevated growth rate in the first 3 days and returned to nearly 90% of the original weight after 7 days (Figures 1B–D). During liver regeneration, ALT (Figure 1E), AST (Figure 1F), and AKP (Figure 1G) all showed a significant increase at the early stage and returned to normal after 72 h. Serum glucose (Figure 1H) was reduced after PHx. Serum triglyceride (Figure 1I) and total cholesterol (Figure 1J) showed a slight decrease at 6 h after 2/3 PHx, increased at 36 and 72 h after 2/3 PHx, and fell at the late phase of liver regeneration. Total bile acids (TBA) (Figure 1K) in the serum significantly increased after 2/3 PHx. PCNA staining on the livers of sham-operated mice and the livers of mice following operation revealed apparent DNA replication, and there were most positive cells at 36 h after 2/3 PHx (Figure 1L).

### GC/MS Chromatograms and Overview of the Metabolomics Data

**FIGURE 2:**
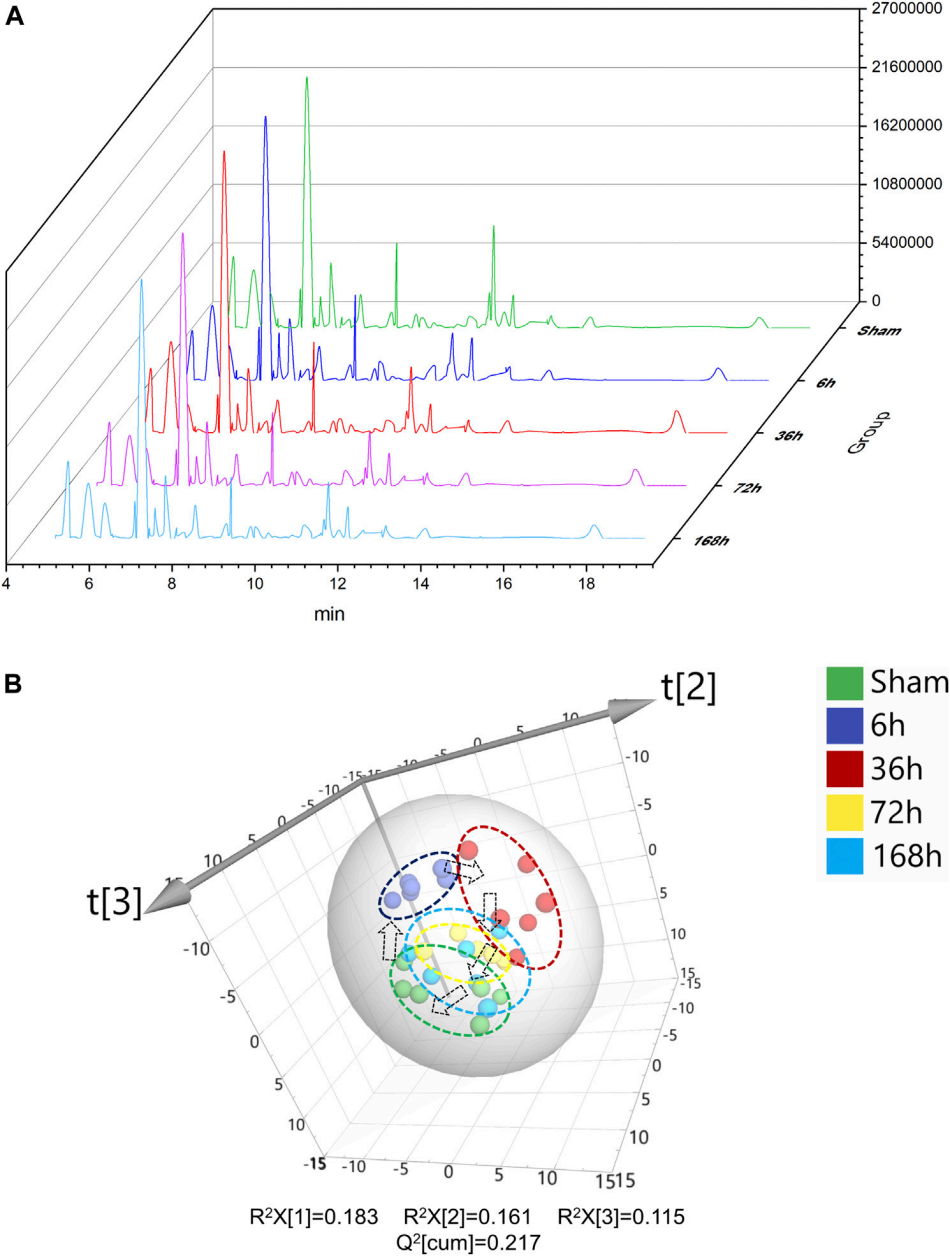
**(A)** Typical GC/MS chromatograms of serum from Sham group and 6, 36, 72, and 168 h after PHx. **(B)** 3D scoress plot of principal components analysis of mouse serum from Sham group, 6 h group, 36 h group, 72 h group, and 168 h group, respectively. Each point represents a metabolite profile of a biological replicate.

Typical serum GC/MS chromatograms from each time point after PHx are shown in Figure 2A. One hundred eighteen compounds were identified, including organic acids, amino acids, carbohydrates, purines and fatty acids, the representative mass spectrum, and the comparison with mass spectrum in the library were shown in Supplementary Data Sheet S1. Unsupervised principal component analysis (PCA) was applied to gain an overview of the metabolomics data. From the scatter plot (Figure 2B), no outlier was found in the PCA analysis. A clear separation between the 6 h group, 36 h group, and sham group was observed, whereas the 72 h group and 168 h group were closer to the Sham group; this suggested that PHx induced significant metabolic change at the early stage and returned to normal during the liver regeneration process.

### Comparison of Machine Learning Methods and Selection of Important Features

**FIGURE 3:**
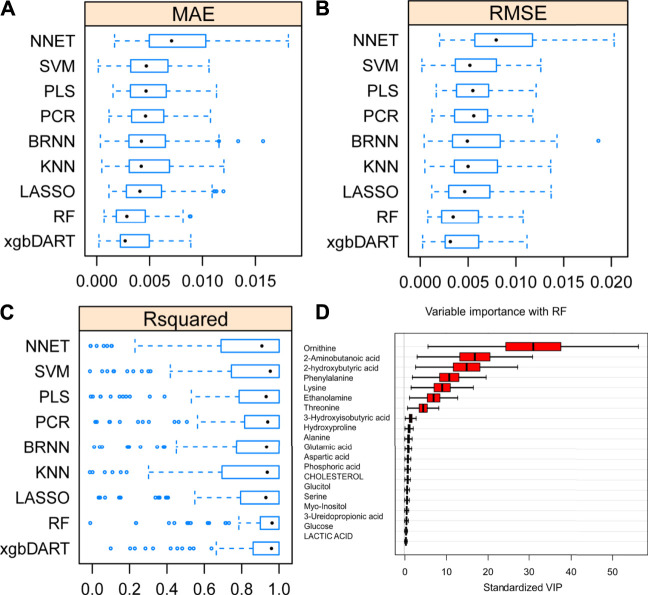
Average MAE **(A)**, RMSE **(B)** and R^2^ **(C)** on 10 repeated 10-fold cross-validation of nine machine learning algorithms for prediction of the liver index from metabolomics data. **(D)** Variable importance revealed by random forest (RF) method.

**FIGURE 4:**
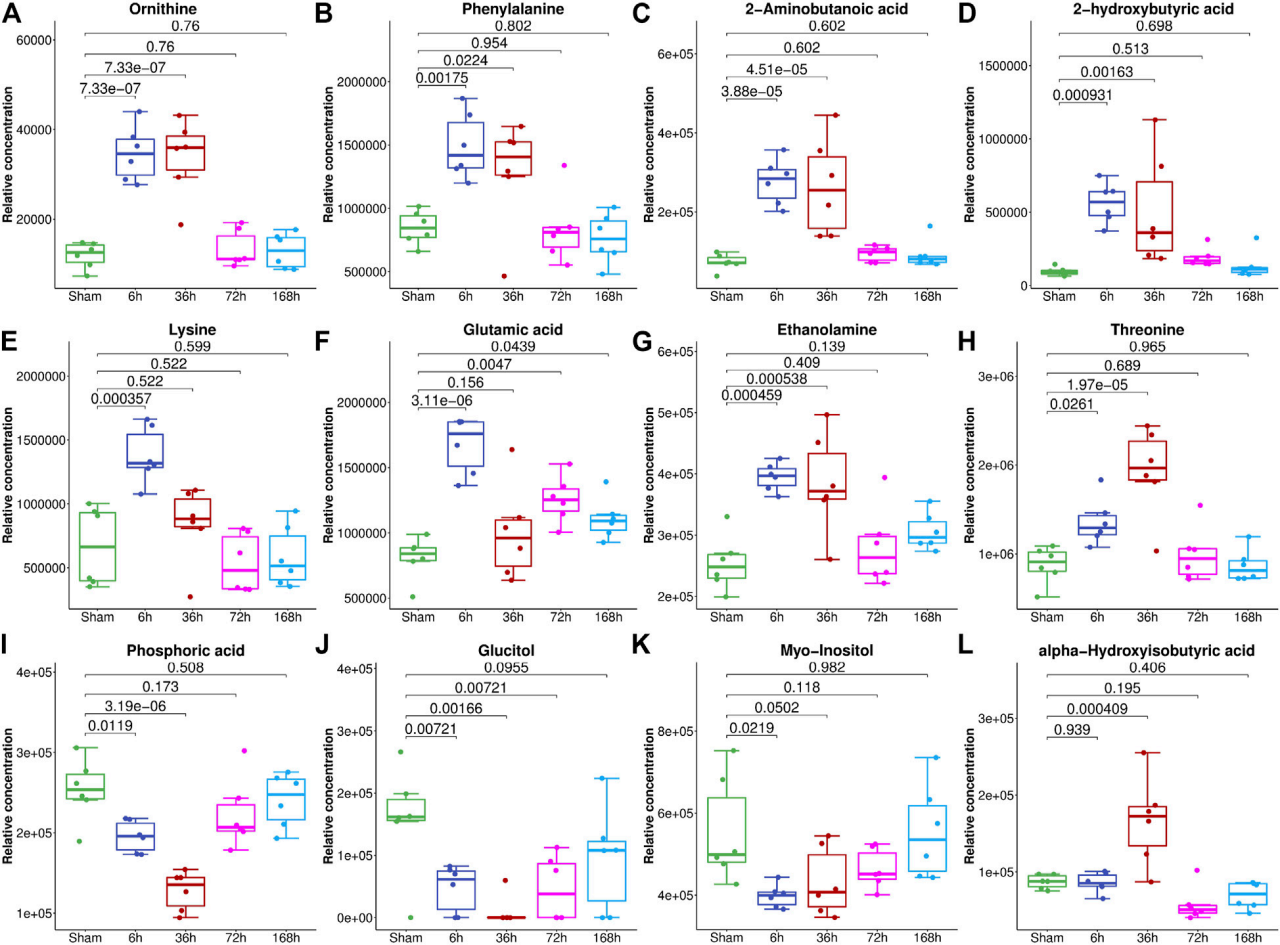
The relative abundance of metabolites with the highest VIP values in the serum of mice from the Sham group and 6, 36, 72, and 168 h after PHx. The box plot shows the relative abundance of metabolites, including ornithine **(A)**, phenylalanine **(B)**, 2-aminobutanoic acid **(C)**, 2-hydroxybutyric acid **(D)**, lysine **(E)**, glutamic acid **(F)**, ethanolamine **(G)**, threonine **(H)**, phosphoric acid **(I)**, glucitol **(J)**, myo-inositol **(K)** and alpha-hydroxyisobutyric acid **(L)**. Data were represented as mean ± S.D.

To select the most suitable machine learning model of the regression between the liver index and metabolites, we performed and compared nine machine learning methods: LASSO, PLS, PCR, KNN, SVM, RF, xgbDART, NNET, and BRNN. We performed 10-fold cross-validation 10 times on the dataset, and MAE, RMSE, and R^2^ were calculated to evaluate the model performance. As shown in Figures 3A–C, the tree-based methods RF method and xgbDART method had the minimum average MAE, RMSE, and the maximum average R^2^. xgbDART method is rather time-consuming and showed no obvious superiority over the RF method; thus, we selected the RF method for further analysis. To choose the most important metabolites contributing to the RF model, we performed VIP analysis and the metabolites which ranked top 20 were selected. Ornithine, phenylalanine, 2-aminobutanoic acid, 2-hydroxybutyric acid, and lysine had the highest VIP values (Figure 3D). The relative amounts of these metabolites were shown in Figure 4A–L.

### Pathway Analysis

**FIGURE 5:**
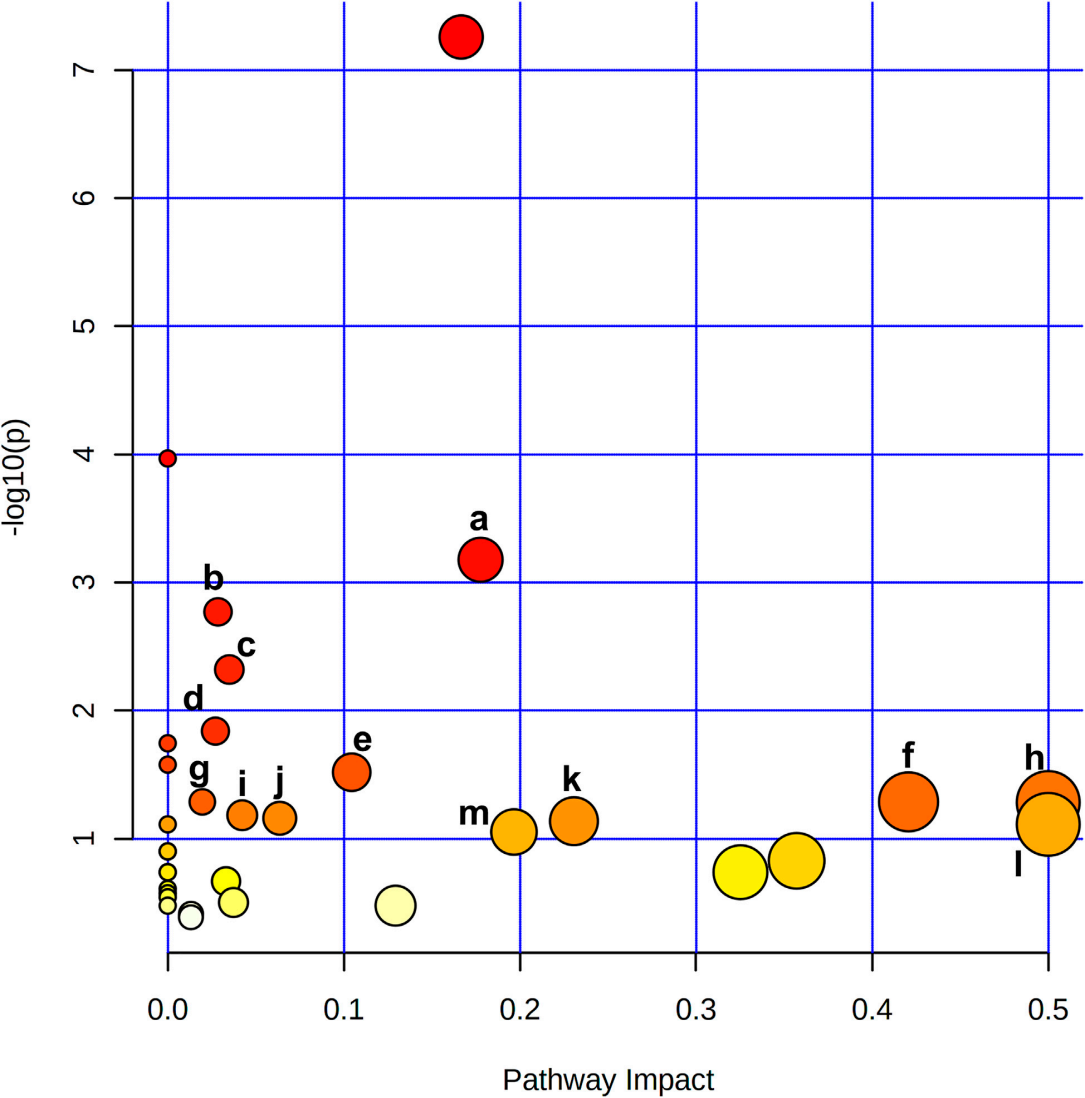
Metabolite pathway analysis based on metabolites displayed significant variation in the serum revealed that a. Arginine biosynthesis, b. Pantothenate and CoA biosynthesis, c. Galactose metabolism, d. Valine, leucine and isoleucine degradation, e. beta-Alanine metabolism, f. Alanine, aspartate and glutamate metabolism, g. Glutathione metabolism, h. Phenylalanine, tyrosine and tryptophan biosynthesis, i. Glyoxylate and dicarboxylate metabolism, j. Cysteine and methionine metabolism, k. Glycine, serine and threonine metabolism, l. D-Glutamine and D-glutamate metabolism, m. Arginine and proline metabolism were the most affected pathways after 2/3 PHx.

To reveal the key pathways changed during liver regeneration, the selected most important metabolites in serum were further analyzed by the online tool MetaboAnalyst (http://www.metaboanalyst.ca). The chosen metabolites were mapped to KEGG metabolic pathways for over-representation and pathway analyses. The pathway was considered to be significantly related which had a *p* value of less than 0.05. Arginine biosynthesis, Pantothenate and CoA biosynthesis, Galactose metabolism, Valine, leucine and isoleucine degradation, and beta-Alanine metabolism, etc. were the most influenced pathways, Figure 5.

### Random Forest Model With a Set of Four Metabolites Were Selected for the Prediction of the Liver Index After 2/3 PHx

**FIGURE 6:**
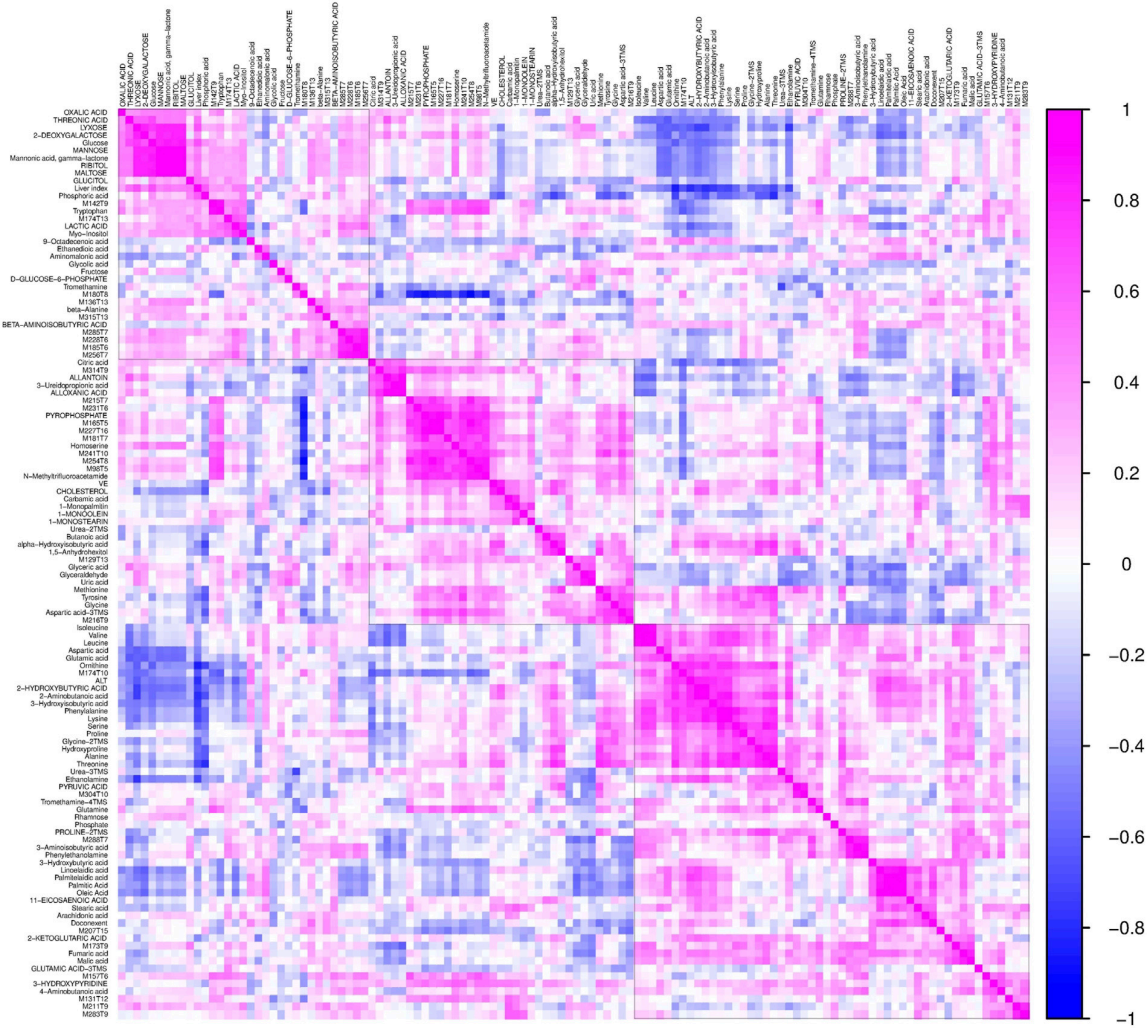
The heatmap shows the correlation coefficients between the liver index and individual metabolites. Each square represents the Pearson’s correlation coefficient between the metabolite of the row and the column. Magenta color represents a positive correlation and blue color represents a negative correlation.

**FIGURE 7:**
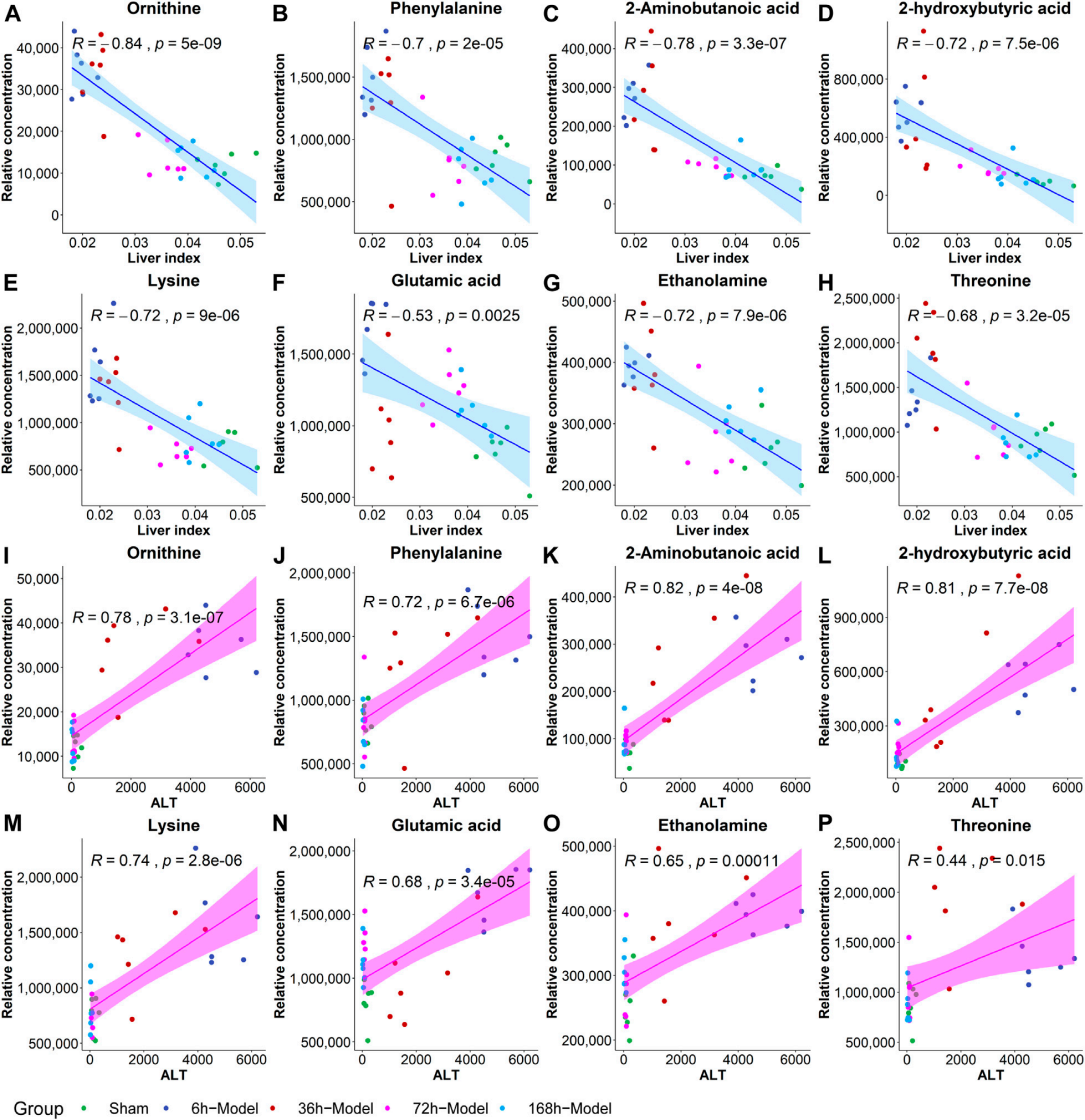
The correlation coefficients between the liver index and metabolites including ornithine **(A)**, phenylalanine **(B)**, 2-aminobutanoic acid **(C)**, 2-hydroxybutyric acid **(D)**, lysine **(E)**, glutamic acid **(F)**, ethanolamine **(G)** and threonine **(H)**, and the correlations between ALT and metabolites including ornithine **(I)**, phenylalanine **(J)**, 2-aminobutanoic acid **(K)**, 2-hydroxybutyric acid **(L)**, lysine **(M)**, glutamic acid **(N)**, ethanolamine **(O)**, and threonine **(P)**.

**FIGURE 8:**
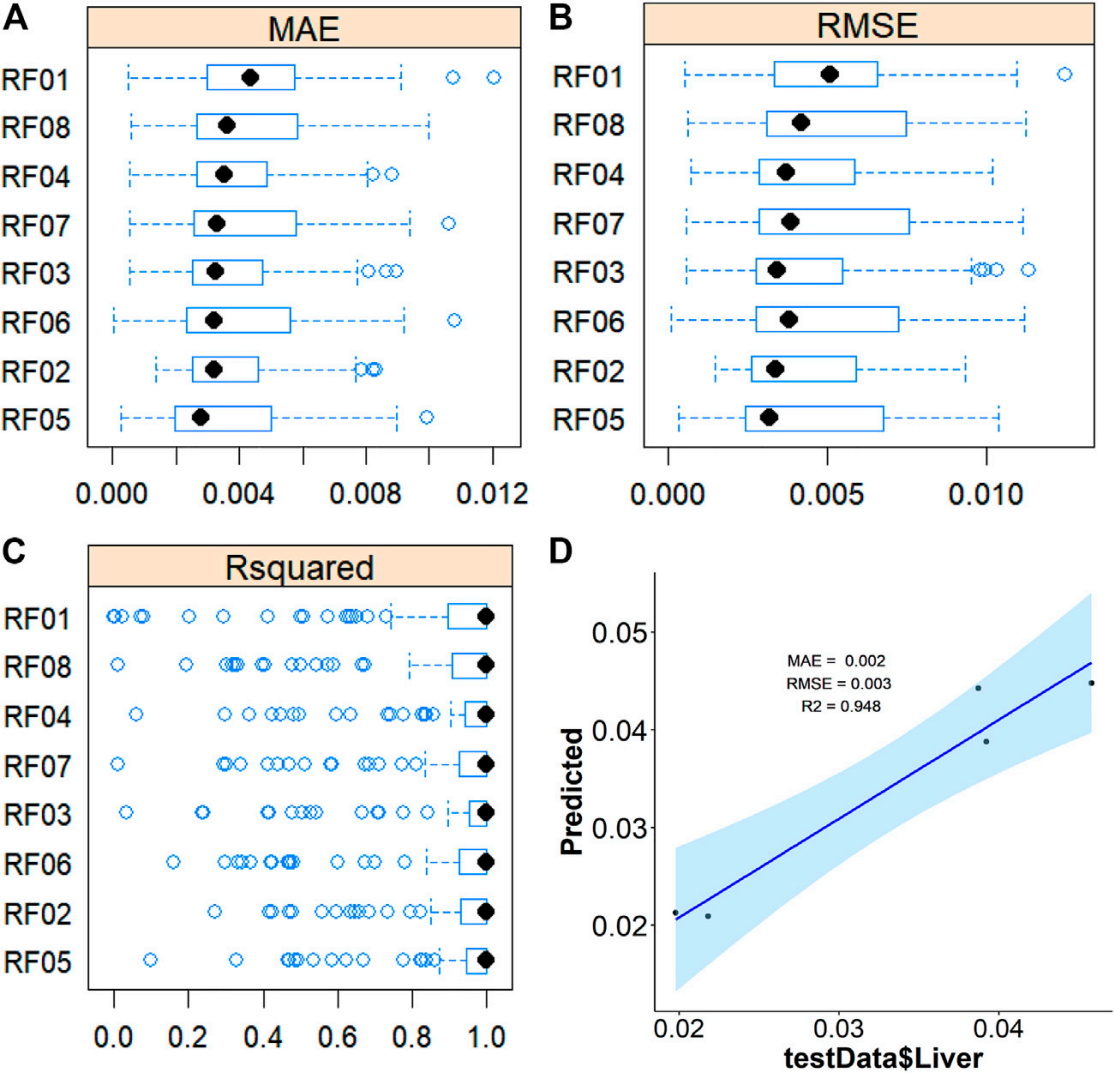
Average MAE **(A)**, RMSE **(B)**, and R^2^ **(C)** on 10 repeated 10-fold cross-validation of random forest method with a different subset of the metabolomics data for prediction of the liverindex from the train data set. **(D)**, the linear regression between the original liver index with the predicted liver index by the RF method in the testing data set.

To further validate the most important metabolites, correlation analysis was performed and shown by heatmap in Figure 6. Metabolites including ornithine (Figure 7A), phenylalanine (Figure 7B), 2-aminobutanoic acid (Figure 7C), 2-hydroxybutyric acid (Figure 7D), lysine (Figure 7E), glutamic acid (Figure 7F), ethanolamine (Figure 7G), and threonine (Figure 7H) all showed an apparent positive correlation with the liver index. They showed obvious negative correlations with ALT, Figures 7I–P. The metabolomics data were partitioned into the training set and testing set, containing 25 samples and five samples, respectively. The comparison of RF methods using a different number of metabolites showed a significant difference in RAE, RMSE, and R_2_ among other models (Figures 8A–C). Then the models were tested on the testing set, and the regression of actual liver index and predicted liver index were performed. The model RF05 containing metabolites ranked top 20 had the minimum MAE and RMSE. Considering the accuracy of prediction with as few metabolites as possible, we selected model RF02 with a set of 4 metabolites including ornithine, phenylalanine, lysine, and 2-hydroxybutyric acid as the final prediction model, and the MAE, RMSE, and R^2^ of the testing set were 0.002, 0.003, and 0.948, respectively (Figure 8D). The metabolic map of these metabolites was shown in Supplementary Figures S1–S4.

## Discussion

After 2/3 PHx, the remnant liver initiates the progress of regeneration and the liver cells would undergo the resting state of the cell cycle (G0) to G1 transition, then S phase, and ultimately mitosis. The progress of liver regeneration includes initiation, progression, and termination, and each of these phases was tightly regulated by numerous signaling pathways (Caldez et al., 2018). To explore the metabolic change and then establish a regression method to predict the liver index at each phase by metabolites in the serum during liver regeneration, we select four time points after 2/3 PHx representing different stages. The liver index at different time points showed a typical growth curve and indicated that these time points could represent the growth of the remaining liver. The serum biochemical indexes representing the liver function and staining results representing the growth of liver cells also indicated the different phases during liver regeneration.

Machine learning has variable applications in healthcare. The main functions of machine learning algorithms include classification, regression, and dimensional reduction. Here we aimed to establish a relationship between metabolites in the serum and liver index at a different time of liver regeneration by regression and select the potential biomarkers of liver regeneration. We compared nine machine learning algorithms for regression, LASSO, PLS, PCR, KNN, SVM, RF, xgbDART, NNET, and BRNN. LASSO is a regression model originally formulated from the linear regression model and performed both for variable selection and regression. PLS and PCR are methods where multivariate data is projected into a smaller coordinate space (dimensional reduction) before regression. SVM method constructs hyperplanes that can be used for classification and regression. RF and xgbDART are both tree-based models which construct a multitude of decision trees. NNET and BRNN are considered deep learning methods and they simulate biological neural networks that constitute animal brains. These methods are more complex non-linear machine learning methods applicable for analyzing high-dimensional metabolomics data. The comparison of MAE, RMSE, and R^2^ of the methods used showed RF and xgbDART are the most accurate methods. xgbDART is much more time-consuming than RF, whereas it offers no significant advantage. Thus we select RF as the method used for further optimization and analysis. From the VIP analysis of RF, we choose different amounts of metabolites to validate the model performance further and evaluate its prediction ability. A metabolite set containing ornithine, phenylalanine, lysine, and 2-hydroxybutyric acid was selected as the potential metabolite set for predicting the liver index after 2/3 PHx.

Networks, including cytokine, growth factor, and metabolic, are the essential circuitry required for liver regeneration (Fausto et al., 2006). Metabolic alteration is proposed to occur immediately after PHx. The previous gene expression data implied that metabolic genes are suppressed during liver regeneration, which is considered paradoxical because it maintains metabolic homeostasis and supports regeneration (Fausto et al., 2006). Currently, there is more understanding of metabolic changes during liver regeneration. Glucose metabolism, lipid metabolism, bile acid metabolism, amino acid metabolism, and one-carbon metabolism are essential for liver regeneration (Huang and Rudnick, 2014; Preziosi and Monga, 2017). We also observed a significant elevation of triglycerides and bile acids and the reduction of glucose in the serum during liver regeneration. It has been reported that glucose supplementation impairs liver regeneration, and preventing the accumulation of hepatic fat also suppresses liver regeneration (Huang and Rudnick, 2014). Dietary caloric restriction accelerates the initiation of regenerative hepatocellular proliferation (Cuenca et al., 2001). These studies revealed the importance of nutrient metabolism in liver regeneration. Bile acids are important for liver regeneration following partial hepatectomy, the extra bile acids cause activation of bile acid receptors including TGR5 and FXR thus preventing hepatotoxicity and providing signals to the regenerative process (Fan et al., 2015; van de Laarschot et al., 2016; Kong et al., 2018). Protein synthesis and amino acid metabolism are essential functions of the liver, and altered amino acid metabolism is observed during liver regeneration. Amino acids are not only components of protein but also work as endogenous signaling molecules. Ornithine is an amino acid that plays a vital role in the urea cycle. A previous study found that urea cycle enzymes were significantly perturbated during liver regeneration, which enhanced urea cycle capacity and increased ammonia elimination (Meier et al., 2019). 2-hydroxybutyric acid is an organic acid derived from alpha-ketobutyrate, and alpha-ketobutyrate is produced by threonine and methionine catabolism and glutathione anabolism. 2-aminobutyric acid is a byproduct of cysteine biosynthesis from cystathionine and it can modulate glutathione homeostasis (Irino et al., 2016). Glutathione is a critical intracellular antioxidant and participates in many critical cellular functions including defense against toxins and free radicals, modulation of cell cycle, and maintenance of immune system homeostasis. Previous literature reported that glutathione, oxidized glutathione, and cysteine levels were doubled after PHx (Huang et al., 1998). Further study confirmed that glutathione plays a role in hepatic NF-κB activation *in vivo* and is necessary for the accurate timing of liver regeneration (Riehle et al., 2013). Urea cycle disorder was reported to be associated with a reduced level of glutathione, increased superoxide radical, and diminished activity of antioxidant mechanisms that may lead to cell damage. We found endogenous metabolites including ornithine, 2-hydroxybutyric acid, and 2-aminobutyric acid, the metabolites involved in the urea cycle and glutathione metabolism, all showed significant change during liver regeneration, and this may be associated with the down-regulated expression of glutamine synthase enzyme and specific activities of urea cycle metabolic pathways (Huang and Rudnick, 2014), and the cytochrome P450 system was down-regulated (Solangi et al., 1988). However, the precise mechanisms behind remain to be verified by further research.

There remain some shortages in this study. Firstly, four time points were selected to represent the initiation, progression, and termination phase of liver regeneration. More time points constituting a complete curve should be evaluated to establish the mathematical model and accurately predict liver weight. Secondly, due to the limitation of GC/MS, many metabolites had not been measured; further use of LC-QTOF/MS is essential to cover more metabolites. Thirdly, a mechanism study to reveal the change of metabolic pathways should be performed. Last but not least, although our model showed good performance in mice, there remains a gap between animals and humans; thus, the transformation from mouse to human should be considered for benefit in the clinic.

## Conclusion

In conclusion, by using a high-throughput GC/MS-based metabolomics technology and machine learning algorithms, we establish mathematical models of liver index and metabolites to predict liver regeneration after 2/3 PHx and compared their performance. We finally choose a time-saving RF method and a set of 4 metabolites containing ornithine, phenylalanine, lysine, and 2-hydroxybutyric acid as a metabolic clock for the accurate prediction of liver index during liver regeneration. Glucose metabolism and amino acid metabolism pathways, including Arginine biosynthesis, Pantothenate and CoA biosynthesis, Galactose metabolism, Valine, leucine, and isoleucine degradation and beta-Alanine metabolism were the most influenced pathways. In the future, we are planning to utilize LC-QTOF/MS based metabolomics to cover more metabolites, and liver regeneration under different circumstances in animals and humans will be performed to validate our model and transform the model into clinic.

## Data Availability

The raw data supporting the conclusion of this article will be made available by the authors, without undue reservation.

## Supplementary Material

Supplementary Data Sheet S1 The comparison of the representative mass spectrum with mass spectrum in the library.

Supplementary Data Sheet S2 Original data of metabolomics and VIPs in each model.

Supplementary Figure S1 The code of machine learning models.

Supplementary Figure S2 The metabolic pathway of ornithine: arginine and ornithine metabolism.

Supplementary Figure S3 The metabolic pathway of ornithine: glutathione metabolism.

Supplementary Figure S4 The metabolic pathway of phenylalanine and lysine: tropane, piperidine, and pyridine alkaloid biosynthesis.

Supplementary Figure S5 The metabolic pathway of 2-hydroxybutyrate: propanoate metabolism.
