# Supplementary figures and images for: Prediction of Liver Weight Recovery by an Integrated Metabolomics and Machine Learning Approach After 2/3 Partial Hepatectomy
Source: Front Pharmacol. 2021 Nov 30;12:760474. doi: 10.3389/fphar.2021.760474 (PMC8669962; doi:10.3389/fphar.2021.760474)

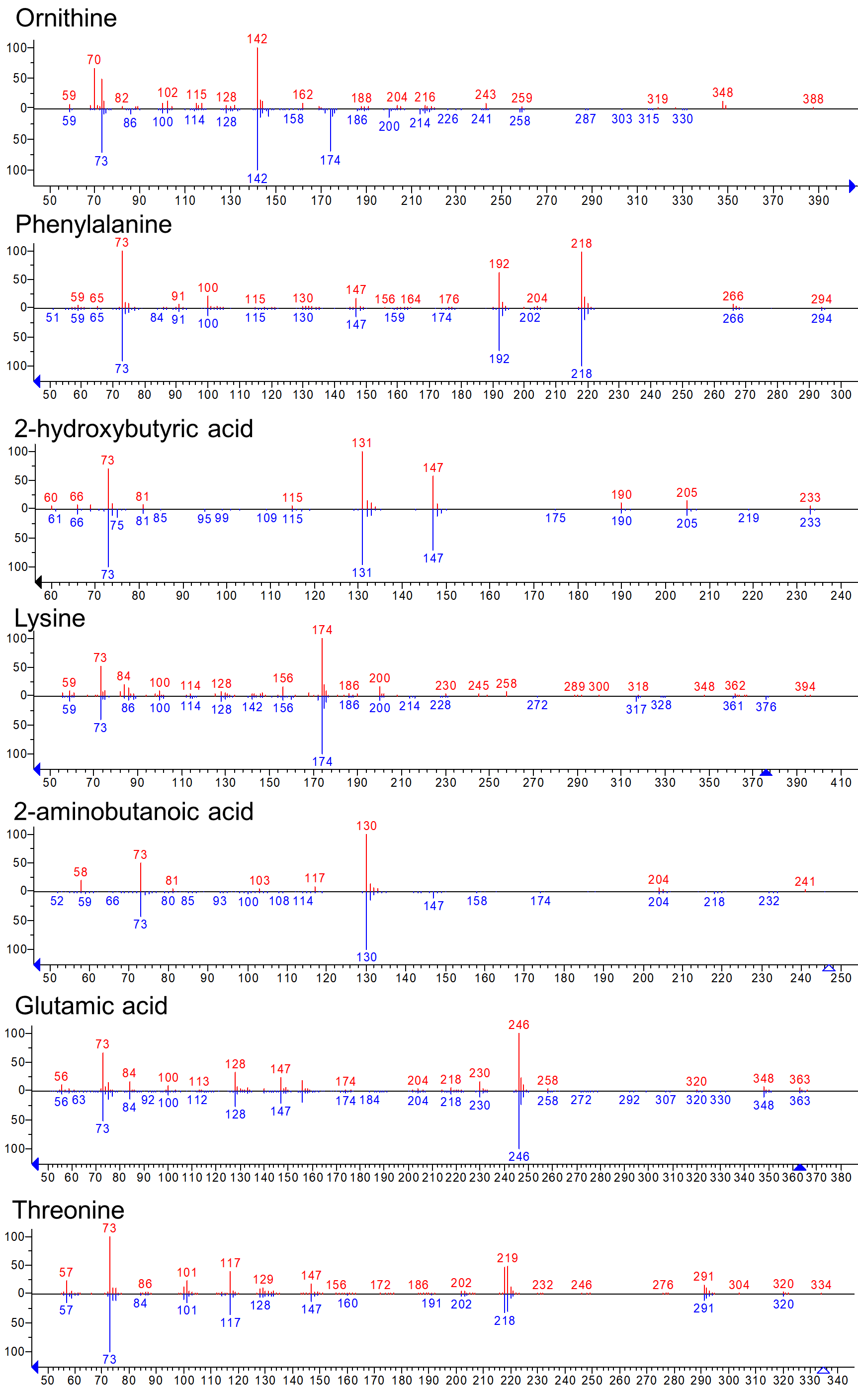

Supplement: Supplementary file 4 [file Image1.TIF]
