# Supplementary material for: Prediction of Liver Weight Recovery by an Integrated Metabolomics and Machine Learning Approach After 2/3 Partial Hepatectomy
Source: Front Pharmacol. 2021 Nov 30;12:760474. doi: 10.3389/fphar.2021.760474 (PMC8669962; doi:10.3389/fphar.2021.760474)
Supplement: Supplementary file 5 [file DataSheet2.docx]

1. **Machine learning method comparison and selection**

library(caret)

library(openxlsx)

library(tidyverse)

library(reshape2)

a <- read.csv("C:\\Users\\Administrator\\Desktop\\ML.csv", check.name = FALSE)

a$Group <- make.names(a$Group)

str(a)

for(i in 3:ncol(a))

{

a[,i] <- scale(a[,i])

}

set.seed(100)

trainData <- a

x = trainData[, 3:ncol(a)]

y = trainData$Liver

fitControl <- trainControl(

method = 'repeatedcv', # k-fold cross validation

number = 10, # number of folds

repeats = 10,

savePredictions = 'final', # saves predictions for optimal tuning parameter

)

setwd("C:\\Users\\Administrator\\Desktop")

dir.create(paste("C:\\Users\\Administrator\\Desktop","\\","ML", sep=""))

setwd("C:\\Users\\Administrator\\Desktop\\ML\\")

Models <- c("LASSO", "PLS", "PCR", "KNN", "SVM", "NNET", "BRNN", "RF", "xgbDART")

models <- c("lasso", "pls", "pcr", "knn", svmRadial", "nnet", "brnn", "rf", "xgbDART")

model_fits <- list()

for(i in 1:length(models))

{

print(models[[i]])

set.seed(100)

model_fits[[i]] = train(x, y, models[[i]], tuneLength=10, trControl = fitControl)

}

names(model_fits) <- Models

model_VIPs <- list()

for(j in 1:length(models))

{

model_VIPs[[j]] <- varImp(model_fits[[j]])

}

model_fitteds <- list()

stats_fitteds <- list()

for(k in 1:length(models))

{

model_fitteds[[k]] = predict(model_fits[[k]], trainData[, 3:ncol(a)])

stats_fitteds[[k]] <- data.frame(

RMSE = round(caret::RMSE(model_fitteds[[k]], y),3),

MAE = round(caret::MAE(model_fitteds[[k]], y),3),

Rsquare = round(caret::R2(model_fitteds[[k]], y),3)

)

}

#VIP

xlsxlist <- list()

for(i in 1:length(models))

{

xlsxlist[[i]]<- model_VIPs[[i]]$importance

}

names(xlsxlist) <- Models

write.xlsx(xlsxlist, "varimp.xlsx",

quote = TRUE,

row.names = TRUE,

fileEncoding = "UTF-8")

models_compare <- resamples(model_fits)

summary(models_compare)

scales <- list(x=list(relation="free"), y=list(relation="free"))

bwplot(models_compare, scales=scales)

Time <- as.data.frame(array(,dim=c(length(models),3)))

for (i in 1:length(models))

{

Time[i,] <- model_fits[[i]]$times[[1]][c(1:3)]

}

names(Time) <- names(model_fits[[1]]$times[[1]])[c(1:3)]

row.names(Time) <- Models

write.csv(Time,file="Time.csv",

quote = FALSE,

row.names = TRUE,

fileEncoding = "UTF-8")

1. **Selection of metabolites in RF method .**

library(caret)

library(openxlsx)

library(tidyverse)

library(reshape2)

a <- read.csv("C:\\Users\\Administrator\\Desktop\\ML.csv", check.name = FALSE, stringsAsFactors=TRUE)

a$Group <- make.names(a$Group)

a <- as.data.frame(a)

str(a)

for(i in 3:ncol(a))

{

a[,i] <- scale(a[,i])

}

set.seed(100)

trainRowNumbers <- createDataPartition(y=a$Group, p=0.8, list=FALSE)

trainData <- a[trainRowNumbers,]

testData <- a[-trainRowNumbers,]

X <- list()

X[[1]] = trainData[c(75)]#Ornithine

X[[2]] = trainData[,c(75,67,87,12)]#VIP top 4

X[[3]] = trainData[,c(75,67,87,12,19,44,84,28)]#VIP top 8

X[[4]] = trainData[,c(75,67,87,12,19,44,84,28,59,10,17,40)]#VIP top 12

X[[5]] = trainData[,c(75,67,87,12,19,44,84,28,59,10,17,40,57,66,112,98,22,90,80,6)]#VIP top 20

X[[6]] = trainData[,c(75,67,87,12,19,44,84,28,59,10,17,40,57,66,112,98,22,90,80,6,82,105,14,91,13,76,61,99,89,58,79,8,115,65,69,35,63,62,111)]#VIP>1

X[[7]] = trainData[,c(75,67,87,12,19,44,84,28,59,10,17,40,57,66,112,98,22,90,80,6,82,105,14,91,13,76,61,99,89,58,79,8,115,65,69,35,63,62,111,68,31,120,117,86,51,93,92,78,33,106,21,37,119,34,83,104,108,53,3)]

X[[8]] = trainData[,c(3:80, 82:119)]

y = trainData$Liver

PredX <- list()

PredX[[1]] = testData[c(75)]#Ornithine

PredX[[2]] = testData[,c(75,67,87,12)]#VIP top 4

PredX[[3]] = testData[,c(75,67,87,12,19,44,84,28)]#VIP top 8

PredX[[4]] = testData[,c(75,67,87,12,19,44,84,28,59,10,17,40)]#VIP top 12

PredX[[5]] = testData[,c(75,67,87,12,19,44,84,28,59,10,17,40,57,66,112,98,22,90,80,6)]#VIP top 20

PredX[[6]] = testData[,c(75,67,87,12,19,44,84,28,59,10,17,40,57,66,112,98,22,90,80,6,82,105,14,91,13,76,61,99,89,58,79,8,115,65,69,35,63,62,111)]#VIP>1

PredX[[7]] = testData[,c(75,67,87,12,19,44,84,28,59,10,17,40,57,66,112,98,22,90,80,6,82,105,14,91,13,76,61,99,89,58,79,8,115,65,69,35,63,62,111,68,31,120,117,86,51,93,92,78,33,106,21,37,119,34,83,104,108,53,3)]

PredX[[8]] = testData[,c(3:80, 82:119)]

fitControl <- trainControl(

method = 'repeatedcv', # k-fold cross validation

number = 10, # number of folds

repeats = 10,

savePredictions = 'final', # saves predictions for optimal tuning parameter

)

setwd("C:\\Users\\Administrator\\Desktop")

dir.create(paste("C:\\Users\\Administrator\\Desktop","\\","ML-RF", sep=""))

setwd("C:\\Users\\Administrator\\Desktop\\ML-RF\\")

Models <- c("RF01", "RF02", "RF03", "RF04", "RF05", "RF06", "RF07", "RF08")

models <- rep("rf",times=8)

model_fits <- list()

for(i in 1:length(Models))

{

set.seed(100)

model_fits[[i]] = train(X[[i]], y, "rf", tuneLength=10, trControl = fitControl)

}

names(model_fits) <- Models

model_VIPs <- list()

for(j in 1:length(models))

{

model_VIPs[[j]] <- varImp(model_fits[[j]])

}

#VIP

xlsxlist <- list()

for(i in 1:length(models))

{

xlsxlist[[i]]<- model_VIPs[[i]]$importance

}

names(xlsxlist) <- Models

write.xlsx(xlsxlist, "varimp.xlsx",

quote = TRUE,

row.names = TRUE,

fileEncoding = "UTF-8")

model_predicteds <- list()

stats_predicteds <- list()

for(i in 1:length(models))

{

model_predicteds[[i]] = predict(model_fits[[i]], PredX[[i]])

stats_predicteds[[i]] <- data.frame(

RMSE = round(caret::RMSE(model_predicteds[[i]], testData$Liver),3),

MAE = round(caret::MAE(model_predicteds[[i]], testData$Liver),3),

Rsquare = round(caret::R2(model_predicteds[[i]], testData$Liver),3)

)

}

library(reshape2)

Predicted <- as.data.frame(array(,dim=c(length(models), nrow(testData))))

for(l in 1:length(models))

{

Predicted[l,] <- model_predicteds[[l]]

}

Predicted <- as.data.frame(t(Predicted))

names(Predicted) <- names(model_fits)

Predicted <- cbind(Predicted, testData$Liver)

library(ggpubr)

old_theme <- theme_update(

axis.ticks=element_line(colour="black"),

panel.grid.major=element_blank(),

panel.grid.minor=element_blank(),

panel.background=element_blank()

)

plots <- list()

for(m in 1:length(models))

{

plots[[m]] <- ggplot(Predicted, aes_string(x = "testData$Liver", y = names(Predicted)[[m]])) +

geom_point() +

stat_smooth(method = "lm", col = "blue", fill = rgb(135,206,250,125,maxColorValue=255)) +

geom_text(x = 0.03, y= max(Predicted[[m]]), label =

paste(

"MAE = ",stats_predicteds[[m]][[2]],"\n",

"RMSE =",stats_predicteds[[m]][[1]],"\n",

"R2 =",stats_predicteds[[m]][[3]]

))+

ggtitle(names(model_fits)[[m]]) +

xlab("testData$Liver") +

ylab("Predicted")+

theme(

plot.title = element_text(color="black", size=24, face="bold", hjust = 0.5),

axis.title.x = element_text(color="black", size=20, face="bold"),

axis.text.x = element_text(angle=0, color="black", size=18),

axis.title.y = element_text(color="black", size=20, face="bold"),

axis.text.y = element_text(color="black", size=20),

axis.line = element_line(color="black", linetype = 1)

)

}

plots

for (n in 1:length(plots))

{

file_name = paste(Models[[n]], ".tiff", sep="")

ggsave(filename = file_name, plot = print(plots[[n]]), compression = "lzw")

}

for(m in 1:length(models))

{

file_name = paste("Predicted_", Models[[m]], ".tiff", sep="")

bitmap(file = file_name, type = "jpeg", res = 600)

plot(testData$Liver, model_predicteds[[m]], pch=19, col="blue", cex.axis=2, cex.lab=2, ylab = paste(Models[[m]], "_Predicted"))

abline(lm(model_predicteds[[m]]~testData$Liver))

legend("topleft", inset=.05, text.font=1, cex=1.5, ncol = 2, title = 'Stats', legend = c("RMSE", "MAE", "R2", stats_predicteds[[m]]))

dev.off()

}

for(m in 1:length(models))

{

file_name = paste("Residual_", Models[[m]], ".tiff", sep="")

bitmap(file = file_name, type = "jpeg", res = 600)

par(mfrow=c(2,2))

plot(lm(model_predicteds[[m]]~testData$Liver))

dev.off()

}

models_compare <- resamples(model_fits)

summary(models_compare)

scales <- list(x=list(relation="free"), y=list(relation="free"))

bwplot(models_compare, scales=scales)

df <- list()

for (i in 1:(ncol(model_fits[[1]]$resample)-1))

{

df[[i]] <- as.data.frame(array(,dim=c(100,length(models))))

}

for (i in 1:length(df))

{

for (j in 1:length(models))

{

df[[i]][,j] <- model_fits[[j]]$resample[i]

}

names(df[[i]]) <- Models

df[[i]] <- melt(df[[i]], variable.name="Group",value.name= names(model_fits[[j]]$resample)[[i]])

}

Time <- as.data.frame(array(,dim=c(length(models),3)))

for (i in 1:length(models))

{

Time[i,] <- model_fits[[i]]$times[[1]][c(1:3)]

}

names(Time) <- names(model_fits[[1]]$times[[1]])[c(1:3)]

row.names(Time) <- Models

write.csv(Time,file="Time.csv",

quote = FALSE,

row.names = TRUE,

fileEncoding = "UTF-8")
